# Super-resolution X-ray tomography using deep learning applied to the 3D quantification of defects in lattice structures

**DOI:** 10.1038/s41598-025-20372-4

**Published:** 2025-10-21

**Authors:** Antoine Klos, Luc Salvo, Pierre Lhuissier

**Affiliations:** Univ. Grenoble Alpes, CNRS, Grenoble INP, SIMAP, F-38000 Grenoble, France

**Keywords:** Super-resolution, X-ray tomography, Deep learning, Lattice, Defects inspection, Multi-resolution registration, Metals and alloys, Characterization and analytical techniques, Imaging techniques, Mechanical engineering, Scientific data

## Abstract

Lattices are intrinsically multiscale materials, forming large structures composed of repeated unit cells, while also including relatively small defects such as pores or grooves. Those defects are detrimental to their mechanical properties and must be quantified. X-ray tomography (CT), a 3D non-destructive imaging technique, is an excellent candidate for this task but it is limited by a trade-off between spatial resolution and scan time. Hence, fully imaging the lattice at a resolution providing a clear depiction of the defects of interest is not compatible with its multiscale aspect, as it would require a prohibitive amount of scan time. Recently, deep learning-based super-resolution has shown remarkable advances in improving spatial resolution of low-resolution CT. However, the validation of these super-resolution workflows is often based on visual quality metrics that do not directly assess the ability to capture critical metrics or phenomena relevant to material scientist. The present study aims at redefining image quality from a material science and task-based perspective, and to quantify the measurement uncertainties associated with super-resolution applied to the 3D characterisation of defects in lattice structures. To address this issue, we have designed a comprehensive super-resolution workflow using a mixed-scale dense network, covering data acquisition, preprocessing, and tailored algorithm validation. The method was tested experimentally on a steel lattice produced by laser powder bed fusion. Super-resolution volumes were computed and their quality was assessed from global greyscale data down to the local scale, investigating both key features of interest: porosity and surface roughness. This approach enhances image quality and improves the morphometric depiction of defects, while enabling a significant reduction in scan time, reaching several orders of magnitude. Thus, we demonstrate that defects inspection in multiscale material such as lattices is now feasible within a reasonable timeframe through deep learning-based super-resolution.

## Introduction

Lattice structures are architectured materials combining material and space, configured in such a way as to have unique attributes used for structural lightening, mechanical energy absorption, gradient properties or heat exchanges^1,2^. Their development has recently benefited from the availability of industrial metal additive manufacturing, notably Laser Powder Bed Fusion (LPBF). This technique makes it possible to manufacture complex geometries that would be impractical using subtractive processes. However, printed parts can still embed defects such as pores, cracks, high surface roughness and variation from dimensional tolerance^3,4^. Those can be detrimental to the structure’s mechanical properties and have to be detected and quantified^1,3^. With this aim, X-ray microtomography, a 3D non-destructive imaging technique^5^, is widely used to explore the inner microstructure of materials. This technique relies on the collection of radiographs, also called projections, at regular angle steps around the sample to image. Reconstruction algorithms such as Filtered Back-Projection are then used to obtain a 3D density map of the sample. The quality of the resulting reconstruction is strongly related to: (i) the number of projections defining the angular sampling; (ii) the X-ray exposure time, linked to the photon counts on the detector; (iii) the pixel size (*i.e.* voxel edge length), taking into account the potential geometrical or optical magnification, which is also linked with the spot size and X-ray source power. Hence, X-ray tomography is limited by a trade-off, where scan time and spatial resolution are adversarial. In recent years, national initiatives have emphasised the need to accelerate the design of architectured materials^6^, in particular with the help of 3D *in situ* and high-throughput imaging, for microstructural and mechanical characterisation of materials. However, the challenge lies in one of the lattice’s specificity: their intrinsic multiscale aspect. They can form relatively large structures composed of repeated unit cells^1^, themselves including smaller structures such as elongated struts, which can include defects at the scale of the fused powder. Consequently, an exhaustive imaging of the lattice at the defect’s resolution is not compatible with its multiscale aspect since it would require a prohibitive amount of scan time, which is not suitable with the perspective of high throughput acquisition and *in situ* testing. There are three main ways to reduce the scan time and they are directly related to the aforementioned imaging quality parameters: (i) by reducing the angular sampling, which can generate artefacts in the reconstruction; (ii) by reducing exposures, which will undoubtedly increase the noise level; (iii) by increasing the pixel size, which will degrade the spatial resolution, hence the minimum resolvable feature size. Whichever method, it involves a detrimental loss of information at the defect’s scale that can prevent its quantitative analysis. Dedicated approaches have been proposed to overcome these limitations, such as efficient reconstruction algorithms^7,8^ that can operate with fewer projections, and denoising strategies^9,10^. In terms of spatial resolution, the gain in scan time can be even more significant because of the cubic relation between pixel size and reconstructed voxel (*e.g.* pixel size multiplied by two means that the reconstructed data size is divided by $2^3=8$). Additionally, for laboratory microtomograph equipped with cone beam X-ray source, increasing voxel size can be done by shortening Source-Detector Distance (SDD). This geometrical adjustment increases the photon counts and therefore reduces the scan time for a same noise level^5^. Hence, maximising the pixel size can theoretically reduce the scan time by several orders of magnitude. As main drawback, the quality, and in particular the sharpness, of the reconstructed images will be affected by the partial volume effect and the inherent point spread function of the system, which will cause object boundaries to be smeared out due to structures whose density varies within a voxel^11^.

To compensate for this information loss, super-resolution techniques were developed. They aim to reduce this partial volume effect using advanced interpolation methods^12^ based on prior knowledge about the object materials^11^ for upsampling the image domain. Initially, methods called multiple-image super-resolution enable a high-resolution image to be estimated from several low-resolution images with sub-pixel offsets between them^13^. Nowadays, single-image super-resolution has improved significantly, taking full advantage of the recent deployment of deep learning, which has proven its relevance for this task^14–16^. The medical imaging community is particularly prolific in this field^15–17^, notably due to the X-ray dose mitigation that this method could achieve. The first approach was to perform super-resolution along one dimension, reducing the slice thickness of clinical X-ray tomography (CT)^18^. Then, it has been used to improve 3D image quality or to reduce the dose for lung CT imaging^19,20^ or coronary CT angiography^21^. Studies also showed that super-resolution can improve the quantitativeness of bone material CT^22,23^, and help to obtain highly resolved computational meshes for digital twin preoperative simulation while reducing the dose^24^. It should be noted that super-resolution was also extensively studied for other clinical imaging modalities such as magnetic resonance imaging (MRI)^16,25,26^. For some imaging modalities such as MRI^25^, some CT scanner, focused ion beam-scanning electron microscope (FIB-SEM)^27^ and edge illumination X-ray phase contrast CT^28^, the method was used in order to obtain cubic-voxel resolution from initial anisotropic sampling. In this particular case, self-supervised algorithms were proposed, taking advantage of the anisotropic resolution by enhancing out-of-plane images from high-resolution in-plane images^25,28^. Moreover, when the availability of annotated training data is limited, for example due to data paucity and X-ray dose limitations as in the medical field, the training data was commonly obtained by generating low-resolution images from downgraded high-resolution data^17,24,29,30^. This practice was found to produce models that performed well on synthetic low/high-resolution paired data, but not well enough in the clinical context^17^. Besides, other applications of super-resolution X-ray tomography included rock^31–35^, microfossil^36^, battery materials^37,38^, composite^39^, additive manufacturing components^40^ and biological materials^41^. In fact, the method is not limited to specific fields, but has mainly been applied to relatively homogeneous structure (*e.g.* bone, rock, porous media) when quantification of specific metric of interest was the aim. For example, with regard to bone material, previous works validated their super-resolution algorithm on bone microarchitectural measurements^22,23,42^ and used finite element simulations to assess the prediction of mechanical properties from super-resolved bone images^42^.

A lot of types of neural network architecture were experimented for super-resolution such as convolutional neural networks (CNN)^18,23,24,28,30,42–44^ and generative adversarial networks (GAN)^20,22,26,32,35,36^, all showing really promising results. Nowadays, commercially available super-resolution algorithms dedicated to X-ray tomography are emerging^44,45^ and will certainly enable this technology to be disseminated within the community^40,41^. But for now, due to the lack of a common benchmark, it is impractical to compare the efficiency of different network architectures and super-resolution workflows. Also, these algorithms are sometimes validated on phantoms (*i.e.* synthetic data) or on downgraded ground truth data^17,24,29,30,44^, not always in 3D, and often using only global similarity metrics such as Peak Signal-to-Noise Ratio (PSNR) and Structural Similarity Index Measure (SSIM)^29,30,36^. Whereas for materials scientists, image quality is not ultimately based on visual similarity, but it is generally evaluated on the ability to capture the metrics of interest (*e.g.* pore size and number, roughness, *etc.*) or investigated phenomena (*e.g.* crack initiation, pore coalescence, *etc.*). Researchers have shown the value of super-resolution applied to experimental data with a resolution ratio up to three^44^. Although they went further than a greyscale similarity analysis, they focused on the comparison of global measures such as pore volume distribution and no local discrepancies in pore morphology or surface roughness were investigated.

Hence, the current challenges in optimising super-resolution of X-ray CT are to redefine image quality from a material perspective, and to quantify the measurement uncertainties associated with this method. To address this issue, we have designed a comprehensive workflow, from data acquisition and preprocessing to tailored algorithm validation by quantitative feature recovery analysis. On the one hand, based on previous studies that proposed the use of MSDnet for super-resolution on synthetic data^29^ and a strategy focused on quantification of metrics of interest but only at a global scale^22^, a detailed procedure to obtain trainable data for super-resolution is proposed. It encompasses a multiscale scanning strategy and a detailed practical method for a high precision multi-resolution registration that includes non-rigid corrections. This registration step was crucial to ensure that the model learned accurate voxel-to-voxel correspondences and cross-scale relationships, rather than capturing positional noise. The approach was tested on experimental X-ray tomography of a steel lattice. On the other hand, super-resolution volumes were computed and their quality was assessed for the first time from global greyscale data down to the local scale, investigating both key features of interest: porosity and surface roughness. Finally, the relevance of the methodology was evaluated by estimating the savings made in terms of scan time, data storage and energy.

## Methods

**Fig. 1:**
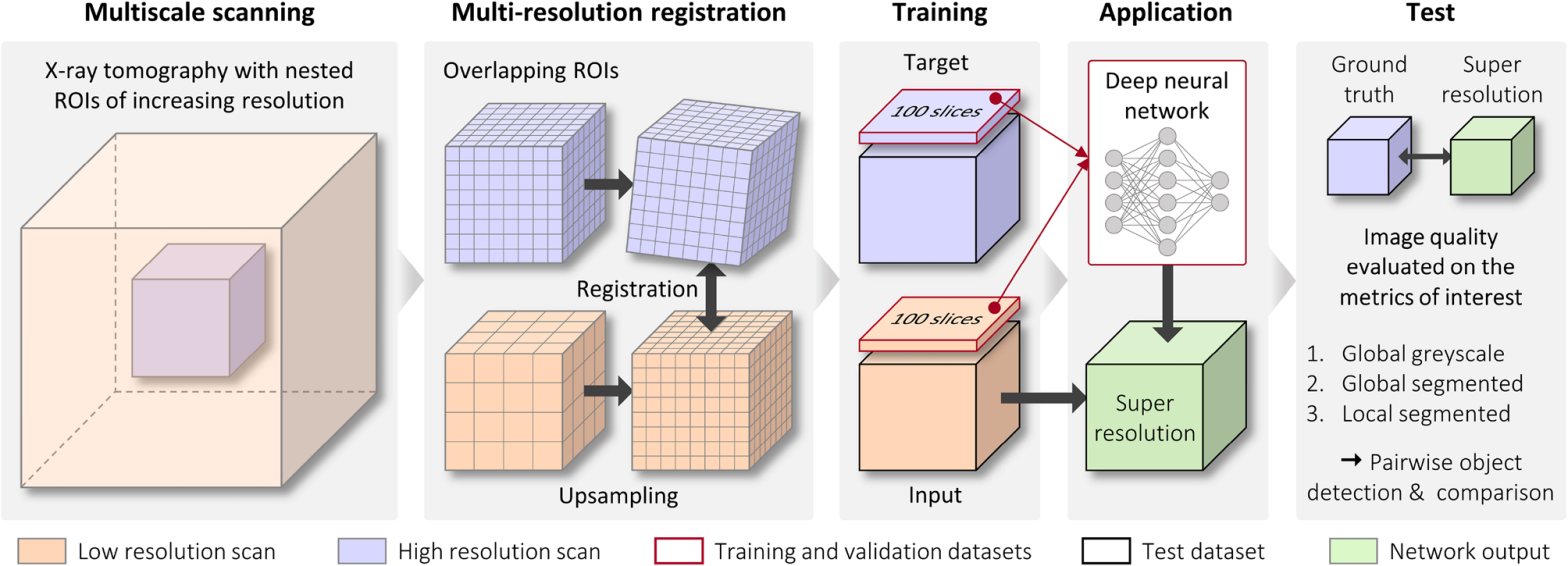
General workflow for 3D super-resolution using deep learning.

The general methodology of this work is illustrated in Fig. 1. First, a multiscale X-ray tomography is performed. It involves scanning nested regions of interest (ROIs) with enhanced resolutions. Then a multi-resolution registration is computed between the ROIs in order to match the 3D images. A part of this registered data is fed into a deep neural network, and the other part is used as a test dataset, to which the trained network is then applied to obtain the super-resolution image. Next, the algorithm is evaluated by comparing the super-resolution image with the high-resolution image from the multiscale tomography. This evaluation is made at different levels such as global greyscale (image similarity metrics), global segmented (volume fraction, feature morphological metrics) and local segmented (pairwise feature detection and comparison). Each step is detailed sequentially in the following sections.

### Experimental developments

#### Sample preparation

The sample was a cuboid ($10\times 10\times 10$ mm) diamond-like lattice made of 316L steel, with strut diameter around 200 ${\upmu }$m. It was printed by Laser Powder Bed Fusion (LPBF) (Orlas Creator®, O.R. Lasertechnologie, Germany) with suboptimal parameters with the aim to maximise the appearance of defects and especially the presence of pores and surface roughness. Laser power was set to 125 W with a spot size of 40 ${\upmu }$m and a motion speed of 800 $\hbox {mms}^{-1}$. The layer thickness was set at 25 ${\upmu }$m and the hatch distance at 50 ${\upmu }$m. The strategy for each layer was to perform one contour trajectory followed by hatching with an angle of $45^{\circ }$ between layers. The powder mean diameter was between 15 and 50 ${\upmu }$m, with a median diameter of 32 ${\upmu }$m (supplier data).

#### X-ray tomography acquisition and reconstruction

A region of interest (ROI) of the sample was scanned with a laboratory microtomograph (EasyTom XL Ultra, RX-Solutions, France) at six different pixel sizes (18 ${\upmu }$m, 9 ${\upmu }$m, 6 ${\upmu }$m, 4.6 ${\upmu }$m, 3.6 ${\upmu }$m and 3 ${\upmu }$m), with the 3 ${\upmu }$m scan being defined as the ground truth (GT) of the study. The pixel sizes were chosen in order to obtain magnification ratios between the GT and other scans about $\times 6$, $\times 3$, $\times 2$, $\times 1.5$, $\times 1.2$, respectively. The ROIs were nested so that the ROI of the GT overlapped with ROIs of all lower resolution scans. Each scan consisted of collecting 1440 projections on a continuous 360 degree rotation of the sample. The microtomograph is equipped with a transmission X-ray source with LaB$_6$ cathode material (L10711-03, Hamamatsu Photonics, Japan) with a spot size below 2 ${\upmu }$m, and a flat panel detector (PaxScan® 2520DX, Varian Medical Systems, USA). The X-ray source voltage was set to 100 kV and its current to 50 ${\upmu }$A, and a 0.27 mm thick aluminium filter was used. More acquisition parameters are given in Table 1. *Xact* (RX Solutions, France) software was used to reconstruct the volumes using a Filtered Back-Projection (FBP) based algorithm for cone beam geometry, with a Tukey filter. No additional filtering or correction was applied to the data.

**Table 1:** Variable acquisition parameters of the multiscale X-ray tomography scans.

Pixel size [${\upmu }$m]	ROI	Frame rate [Hz]	SDD [mm]	SOD [mm]
3	$1275\times 1782\times 1782$	0.5	457.7	10.8
3.6	$1243\times 1759\times 1759$	0.66	382.3	10.8
4.6	$1190\times 1713\times 1713$	1.05	298.7	10.8
6	$1125\times 1639\times 1639$	1.75	229.2	10.8
9	$1125\times 1639\times 1639$	1.75	229.2	16.2
18	$1125\times 1639\times 1639$	1.75	229.2	32.5

SDD, source-detector distance; SOD, source-object distance. Each projection was averaged from the accumulation of 5 frames.

### Data preparation and deep learning-assisted super-resolution

#### Multi-resolution registration

**Fig. 2:**
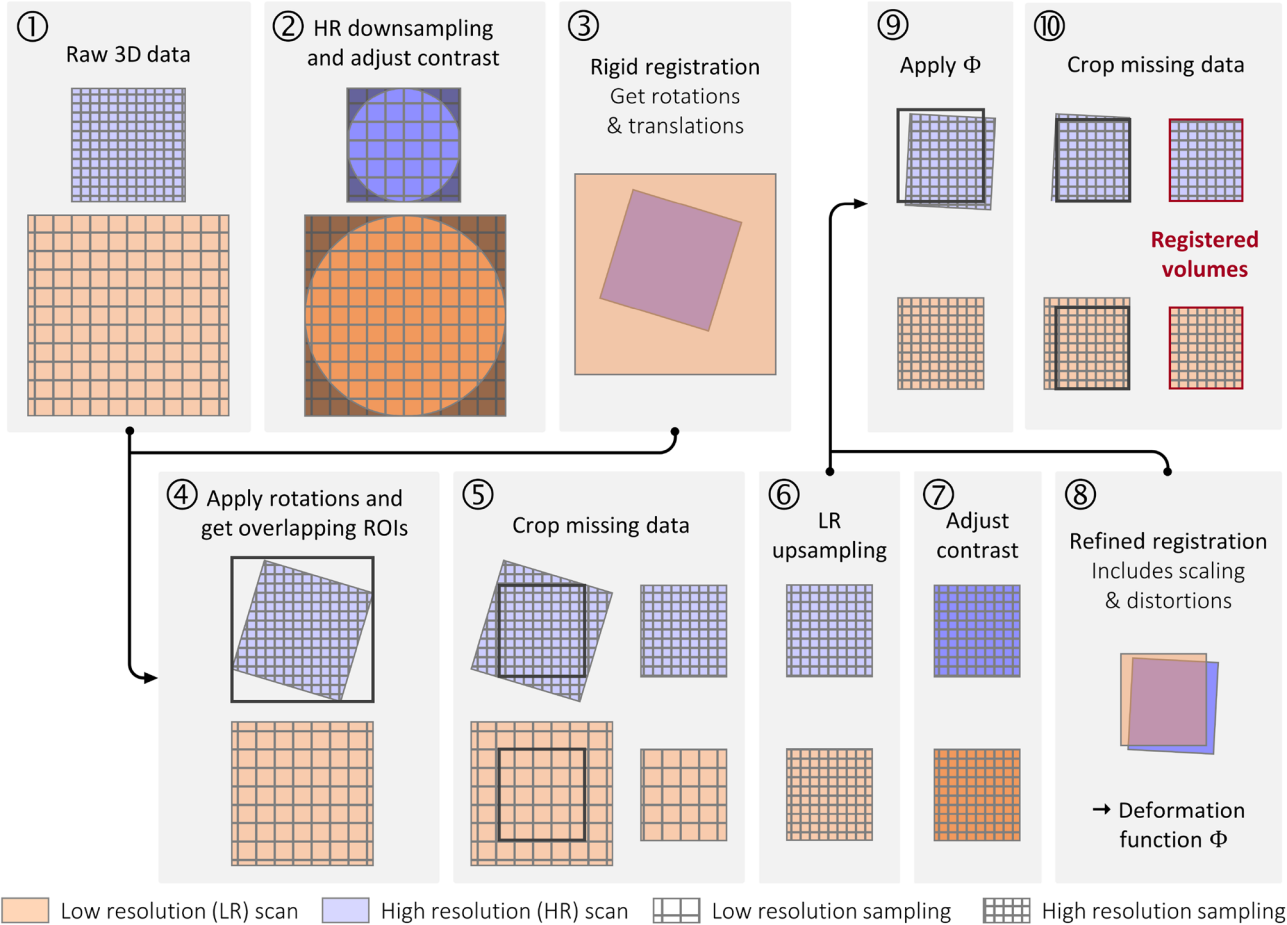
Procedure of multi-resolution registration in the case of working with two nested ROIs of increasing resolution.

A multi-resolution registration procedure was developed based on the volumetric correlation software *Spam*^46^. A scheme of the procedure workflow is presented in Fig. 2 in the case where only two volumes are registered together (step ①).

The first step of the method consisted of matching the voxel size of the volumes, by downsampling (tricubic spline interpolation) them up to the largest voxel size, and maximising the greyscale contrast generating only 1‰ saturated voxels (step ②). To find the overlaps between the ROIs, a coarse and rigid registration was computed with an L2 norm of the difference and applied to the raw data (convergence options: 1,000 maximum iterations, $|\delta \Phi | \le 10^{-3}$, multiscale binning levels [4, 2]), using the encoder position of the microtomograph as initial guess. This step allowed to obtain a first approximation of the translations and rotations between the volumes (steps ③ and ④). Then, ROIs were adjusted by cropping non overlapping regions and potential missing data due to interpolation of points outside the boundaries of the input (step ④ and ⑤). Steps ② to ⑤ were repeated but this time by upsampling the voxel size down to the smallest and by computing a refined non-rigid registration (convergence options: 100 maximum iterations, $|\delta \Phi | \le 10^{-2}$, binning 2) (steps ⑥ to ⑩). The non-rigid component of the transformation resulted from minor scaling effects, caused by imprecise motor position readouts, specifically in the source-to-object distance (SOD) and source-to-detector distance (SDD). Finally, upsampled and registered volumes were obtained. In the case of multi-resolution registration which included more than two different resolutions, the deformation functions $\Phi$ were computed between each pair of intermediate volumes, and a composition of successive functions $\Phi$ was used to retrieve the deformation between the GT and each volumes. Also, a mask corresponding to the tomographic cylinder (*i.e.* the cylinder in which the sampling theorem is satisfied for accurate image reconstruction) was used to restrain the correlation calculation to this region. The cylinder is represented in 2D in Fig. 2, step ②.

#### Deep neural network for super-resolution

Registered 3D images were first cropped to the cuboid inscribed in the tomographic cylinder. Resulting images were divided into training, validation and test datasets. In practice, the first 100 slices ($\approx 10~\%$ of the whole data) along the tomographic axis were devoted to training and validation with a ratio of 80 % and 20 %, respectively. The neural network was a 2.5D Mixed-Scale Dense Network (MSDNet)^47^ with a configuration described by Pelt *et al.*^48^ (100 layers, dilations within the [1,10] interval, ADAM optimiser) that has proved its effectiveness for super-resolution tasks. Slabs of five consecutive slices were used in the 2.5D model to include minimal 3D information while keeping the training as fast as possible for practical applications. Due to the specificity of lattice structures, training and validation dataset were masked to restrain the networks training to data in the bulk material. The mask was generated by applying a Li^49^ threshold on the high-resolution greyscale data, followed by a binary dilation with 3D ball-shaped structuring element with a radius of 5 pixels. The dilation was performed in order to keep the greyscale gradient of the material surface within the training dataset. Slices of the mask are shown in Supplementary Fig. S1. Finally, networks for each resolution pair were trained for about 3,000 epochs (48 hours on one GPU), using an L2 loss (mean squared error loss) to convert lower resolution images (input) to their corresponding high-resolution GT (target) for each pair of input/target 3D images. The model with the lowest validation loss was selected as the best model. The evolution of the validation loss function during training is shown in Supplementary Fig. S2. Trained networks were then applied to the test datasets to obtain the super-resolution images. This approach follows the generic method provided by Hendriksen *et al.*^29^. Both training and application were done using one GPU (NVIDIA Tesla V100 NVLink) on the GRICAD infrastructure, which is supported by Grenoble research communities.

#### Evaluation of the registration sensitivity

In order to evaluate the performance of the multi-resolution registration procedure, a dedicated dataset was created without additional experiment. The already computed transformation matrix of the registration, $\Phi$, was adjusted to apply an additional 3D transformation only to the GT volume (tricubic spline interpolation), combining rotation, scaling, and translation along all axes. The transformations magnitude was set between 0.5 and 5 voxel edge length (pixel size) by calculating the mean voxel displacement in the whole volume. Neural networks were trained using these datasets as GT, to mimic the case of a super-resolution workflow in the absence of a reliable multi-resolution registration procedure.

### Quantification of the quality of the 3D images

The quantification of the image quality was performed at several levels, comparing the improvement of the image quality on: (i) the greyscale data, (ii) the segmentation of the pores at a global and local scale, and on (iii) local and global surface measurements.

#### Global metrics

*Global greyscale* – Peak Signal-to-Noise Ratio (PSNR) and Structural Similarity Index Measure (SSIM) were computed using their implementations in the *scikit-image* package^50^. The parameters of the SSIM computation were adjusted to match the implementation of Wang *et al.*^51^. As for deep neural network training, the same mask was used to avoid greyscale measurements outside the bulk material. Slices of the mask are shown in Supplementary Fig. S1.

*Global segmented* – The two 3D images were first binarised using an Otsu^52^ threshold to discriminate the background (air) from the lattice (steel). A detection threshold $\alpha$ was defined to discard objects with a volume smaller than $3^3$ voxels (with the HR voxel size) because defined as not significant, and the remaining were labelled in each images. Slices of the binary images are shown in Fig. 5a. This simple segmentation process was chosen in an attempt to evaluate only the performance of the super-resolution and not that of the segmentation tool.

Number of objects, mean volume, volume fraction and global Dice score were computed from these labelled images. The sphericity of each pore was calculated using $sphericity = 6 V \sqrt{\pi /S^3}$ with *S* the surface calculated from its mesh computed with a marching cubes algorithm^53^ and *V* the volume contained within the mesh.

#### Local morphometric and performance measurements

**Fig. 3:**
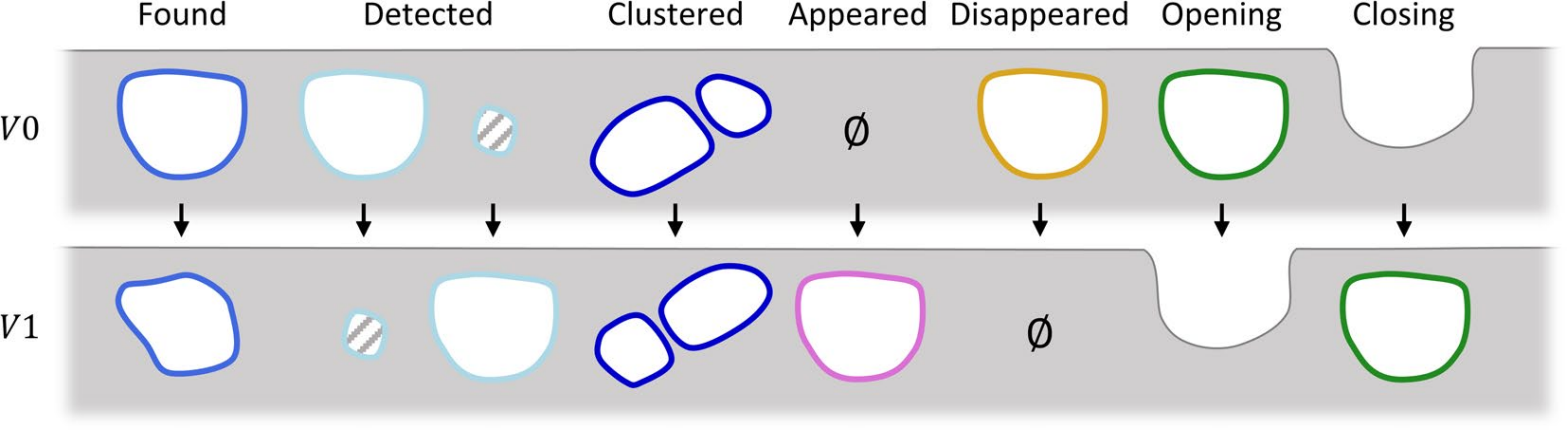
Illustration of pore status as defined by the pairwise object detection algorithm. Grey regions represent the lattice material, white ones the pores and hatched areas the pores with a volume below the detection threshold $\alpha$ of $3^3$ voxels. Pore outline colours are reused in Fig. 6 according to their status. $\emptyset$ stands for the absence of pore.

The aim was to compare each individual pore between two 3D binary images, such as the GT and a super-resolution image. Once pores had been matched in the two volumes, a more in-depth analysis regarding some key morphological parameters such as their volume and sphericity was carried out. For this purpose, we first developed a pairwise object detection algorithm. It allowed to compare two 3D binary images, the ground truth image *V*0 (*i.e.* the segmented high-resolution scan) and a measured image *V*1 (*i.e.* the segmented lower resolution or super-resolution image). Then, in order to be compared two by two, the objects, *i.e.* the pores, from both images had to be paired. For this step, we distinguished between different object statuses that are illustrated in Fig. 3. More details about the statuses can be found in Supplementary Note S1. Besides, more information about the algorithm can be found in Supplementary Fig. S3; in-depth examples are shown in Supplementary Figs. S4 and S5; and the validation of the algorithm on a phantom is presented in Supplementary Fig. S6. The algorithm allowed to extract some metrics such as the rate of “*Disappeared*” pores. It was calculated on the GT data as the ratio between “*Disappeared*” over the union of “*Found*”, “*Clustered*”, “*Detected*” and “*Opening*” pores. Paired pores were also compared in terms of segmentation performances using Sensitivity (true positive rate), Specificity (true negative rate) and Dice score. Also, morphological measurements were performed comparing their volume and sphericity with the procedure defined above in Methods. Moreover, some specific pores characterised by a more complex geometry were extracted together with their matched counterparts in order to be compared qualitatively. For observation only, the pore meshes were rendered with the software *Paraview*, using a 200-iterations smoothing filter to remove the visual staircase effect.

#### 3D roughness and lattice surface comparison

The 3D roughness parameters *Sa*, *Sv* and *Sp* and height maps were computed with the code developed by Steinhilber *et al.* ^54^. The cut-off wavelength considered to discriminate form and waviness from roughness was set to $\lambda _c = 250$ ${\upmu }$m and the S-filter cut-off wavelength was set to $\lambda _S = 15$ ${\upmu }$m as an anti aliasing filter. More details about the choice of $\lambda _c$ can be found in Supplementary Note S2. To avoid edge effects due to the filtering, points closer to the image boundaries than $\lambda _c/2$ were discarded from the computations. Comparison of surface heights between the ground truth lattice (*i.e.* from the high-resolution scan) and the measured lattice (*i.e.* from the lower resolution scan or the super-resolution image) was done with the same code, using the included implementation of the Signed Euclidean Distance Transform. For observation only, the 3D height maps were rendered from surface meshes with the software *Paraview*, using a 200-iterations smoothing filter to remove the visual staircase effect.

### Calculation of scan time, storage and energy savings

Scan time and data storage size were estimated for existing data using the parameters detailed in Table 1, and extrapolated when necessary for comparison purposes. The *original* scan time corresponded to the duration of an equivalent tile scan at the GT pixel size (*i.e.* 3 ${\upmu }$m) while imaging the ROI of the low-resolution scan. It was calculated by multiplying the scan time of the GT by the ratio between the ROIs and taking into account the difference in pixel size. No overlap between tiles was considered. The *super-resolution* scan time corresponded to the GT scan time plus the low-resolution scan time. Using the same approach, the *original* data storage size corresponded to the size of the equivalent tile scan. The *super-resolution* data storage size corresponded to the GT and the low-resolution scan size plus the data size of the super-resolution images that would cover the equivalent tile scan. Both were calculated considering a 16-bit image encoding and taking into account the size of the projection data when applicable. Energy consumption was calculated by taking into account the scan time and the networks training and inference time. The CT-scan consumption per hour was estimated at 5 kWh (internal data). Without usable data for estimating the consumption of the deep learning computations made on GRICAD, the calculation was approximated at 0.482 kWh per hour, using the GPU power consumption of the Jean Zay supercomputer^55^. Training duration was 48 hours and inference around 25 minutes. Consumption for *original* method included only CT-scan consumption for scanning the full ROI at high-resolution whereas for *super-resolution* it included the consumption of the multi-resolution scan plus the one of the network training and inferences for the full ROI.

## Results and discussion

### Multi-resolution registration of a lattice structure

**Fig. 4:**
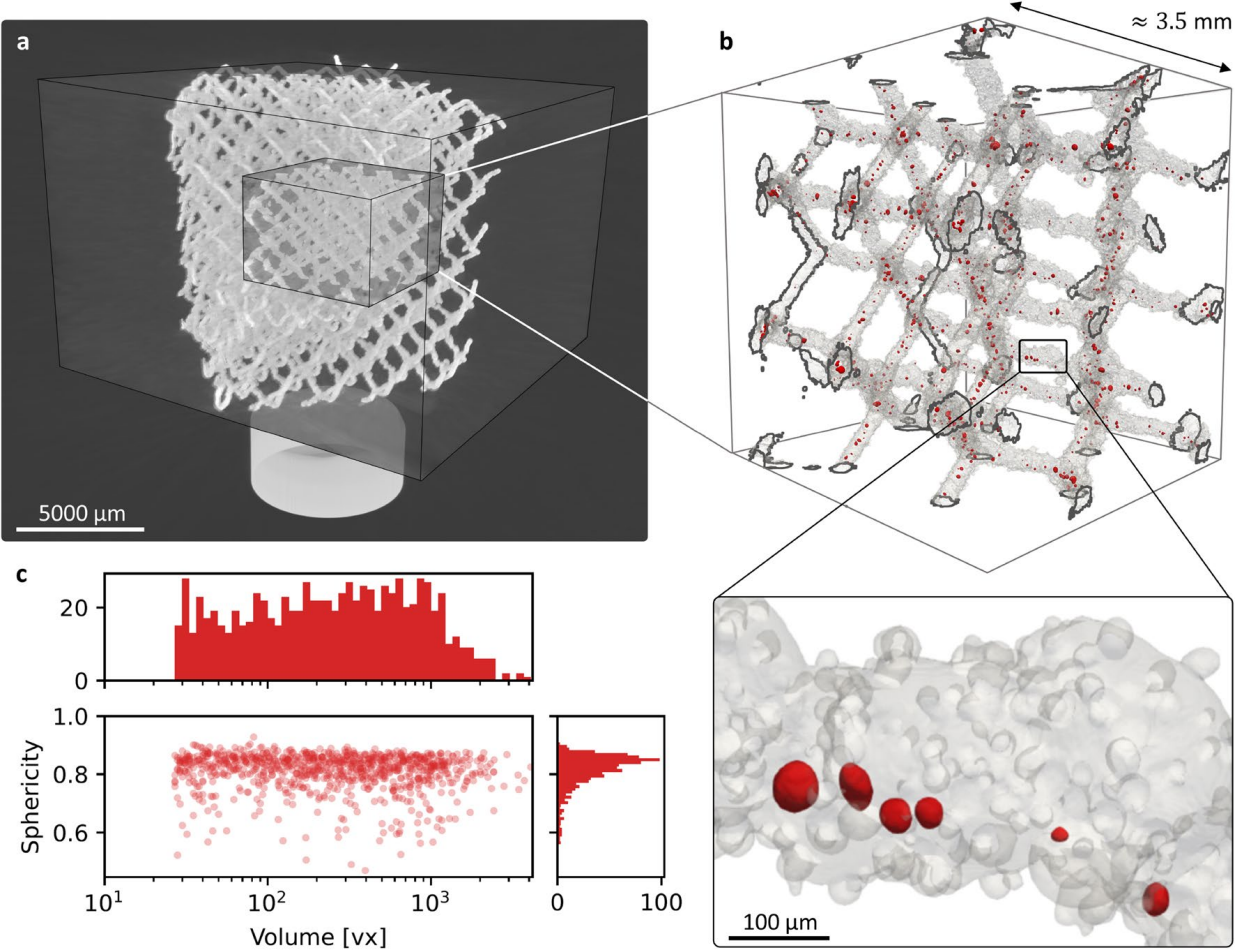
Presentation of the studied lattice. (**a**) 3D rendering of the multi-resolution registration result displaying nested ROIs of increasing resolution. Denser regions are depicted in white. (**b**) (*top*) 3D rendering of the overlapping ROI. The lattice material is rendered in grey with a transparency to display the pores in red. The darker contours represent the ROI boundaries that intersect with the lattice. (*bottom*) Zoomed-in inset for visualisation of typical pore shapes and surface roughness. (**c**) Sphericity of each pores according to their volume. Histograms at the sides of the plot show the marginal distributions of both morphological parameters.

The Fig. 4a illustrates the nested ROIs of increasing resolution that were localised in 3D using the strategy of multi-resolution registration described in Methods. The overlapping region, shown in the Fig. 4b, included about eight lattice cells. The 3D rendering emphasises the rough lattice surface due to visible partially melted powders.

As expected, the printed part also displayed numerous pores, highlighted in red in the rendering. The histograms in Fig. 4c exhibits that the pores were rather spherical, with a mean sphericity of 0.81. The pore volume lied between 27 (*i.e.* $\alpha$, the detection threshold) and 4094 voxels (equivalent sphere radius between approx. 2 and 10 pixels in length), with 99 % of pores smaller than the maximum volume of the powder (*i.e.* 2424 voxels for powder diameter of 50 ${\upmu }$m). Hence, the intentional choice of suboptimal printing parameters produced a lattice combining defects such as pores and surface roughness, which will be used as a practical case study to assess the capability of the super-resolution workflow for defect detection and quantification.

### Global quantification of image quality and its limits

Super-resolution images were obtained following the procedure detailed in *Methods*, with the proposed multi-resolution registration approach. We focused on two magnification factors, $\times 6$ and $\times 3$, between the ground truth (GT) and the low-resolution scans with pixel sizes of 9 ${\upmu }$m and 18 ${\upmu }$m, respectively. To simplify the nomenclature, the low-resolution scan at 18 ${\upmu }$m pixel and 9 ${\upmu }$m pixel were named LR-6 and LR-3, respectively, the number corresponding to the magnification factor relative to the GT. Accordingly, the super-resolution images from LR-6 and LR-3 were named SR-6 and SR-3, respectively, and exhibit a voxel size of 3 ${\upmu }$m as the GT. Evolution of the loss function during both trainings is given in Supplementary Fig. S2. As expected, due to the inherent loss of detail in the image compared to GT as pixel size increases, the L2 loss was about an order of magnitude higher for SR-6 than for SR-3, after 48h of training. In a first approach, a global quantification of the obtained image quality was investigated.

#### On greyscale data

**Fig. 5:**
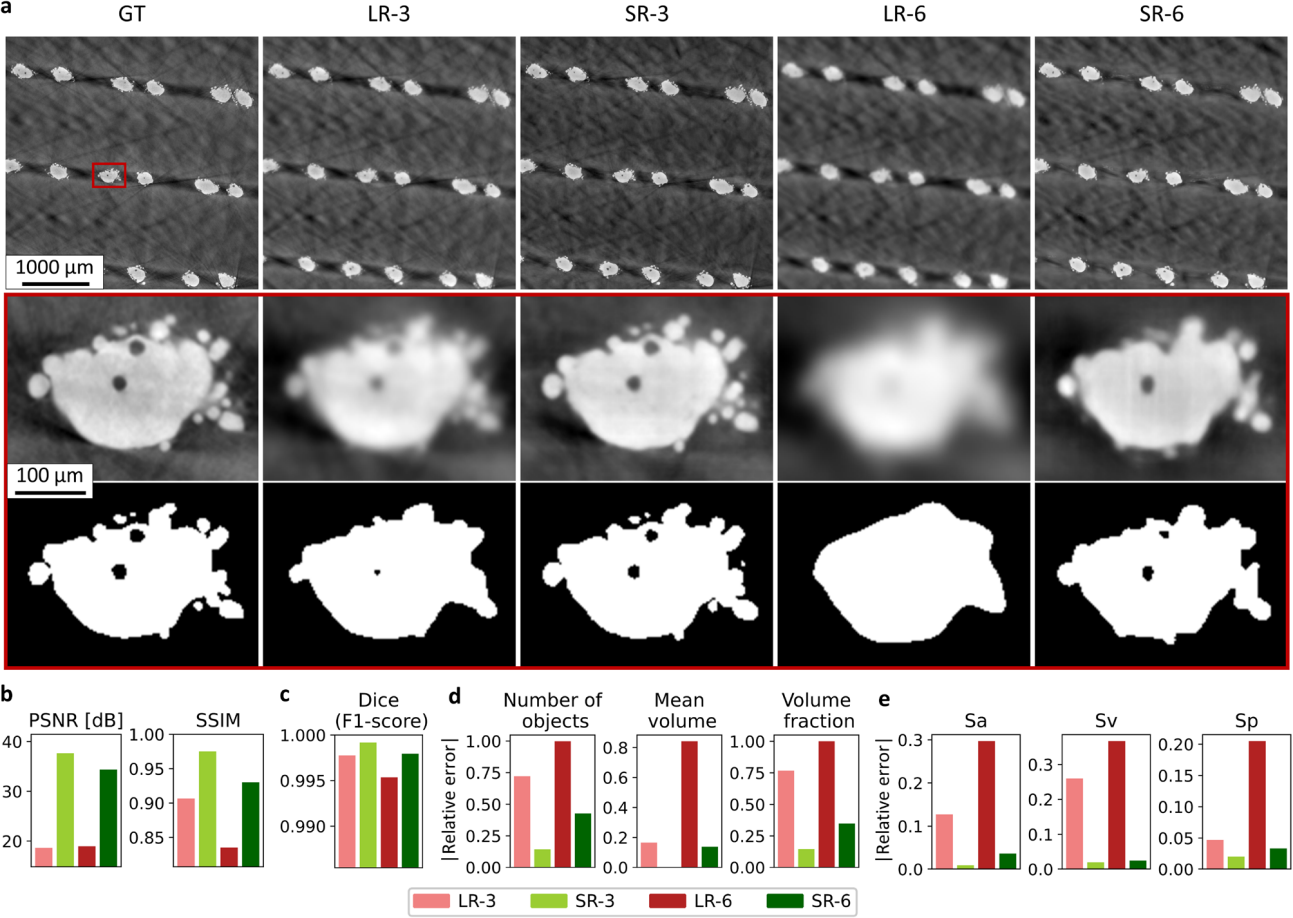
Qualitative and global quantitative assessment of the super-resolution result. (**a**) Horizontal slices of CT-scans with corresponding super-resolution outputs (*top*), with zoomed-in insets (*centre*) and their segmentation (*bottom*). Low resolution images were upsampled to match the sampling of GT using tricubic spline interpolation. Image contrast was extended with minimum and maximum greylevels for better visualisation. (**b**) Global greyscale similarity measurements with GT as the reference. (**c**) Global performance measurement of the segmented images. (**d**) Error quantification of global pore parameters. (**e**) Error quantification of global surface roughness parameters.

Figure 5a shows slices of the different scans and their super-resolution counterparts, in the test dataset.

The zoomed-in insets demonstrate clear improvement of details sharpness and visual similarity between the GT and the super-resolution images. Obviously, the SR-3 appears more similar to the GT compared to the SR-6. This qualitative depiction is validated by quantitative similarity metrics such as the PSNR and the SSIM (see Fig. 5b). Indeed, PSNR of super-resolution images approximately doubled compared to the one of low-resolution images, and the SSIM increased by 8 % and 11 % for SR-3 and SR-6, respectively. It should be noted that super-resolution images also appear to be less noisy than the GT image. Since the low-resolution input images and high-resolution targets images have independent noise, the MSD network also tends to denoise the data as it was demonstrated by Pelt *et al.*^48^. Although the initial images are only slightly noisy, this denoising is beneficial for both image quality and subsequent analysis. It should be noted that MSDnet has also proven its effectiveness in denoising very noisy data in a different architecture called Noise2Inverse^9^.

#### On segmented data

The binary images in Fig. 5a show a smoothing of the lattice surface combined with a disappearance of pores when increasing the pixel size. In contrast, the super-resolution binary images exhibit an better matching with the GT compared to the one of low-resolution scans, both for surface and pores delineation. This observation is confirmed by the measurement of the improved Dice score for super-resolution images (see Fig. 5c). In order to characterise the features of interest (pores and roughness), a global quantification was performed. First, the porosity was assessed regarding the number of pores, their mean volume and their volume fraction (see Fig. 5d). For all parameters, a strong decrease was measured on the error made on such quantification with the super-resolution images and the GT as reference. Moreover, the error made on surface roughness parameter *Sa*, *Sv* and *Sp* was quantified with the same findings (see Fig. 5e).

The quantification at the global scale, both on greyscale and segmented images, demonstrated a clear improvement of the image quality provided by super-resolution. However, such global quantification is not appropriate to assess the super-resolution workflow and algorithms because it does not guarantee against loss of information at the defects scale, which is often the critical information investigated by materials scientist. It is then necessary to deploy a more advanced and task-based validation by focusing at the local scale on both features of interest in this study: the porosity and the surface roughness. The next two sections will be devoted to this topic.

### Super-resolution effect on the 3D quantification of pores

**Fig. 6:**
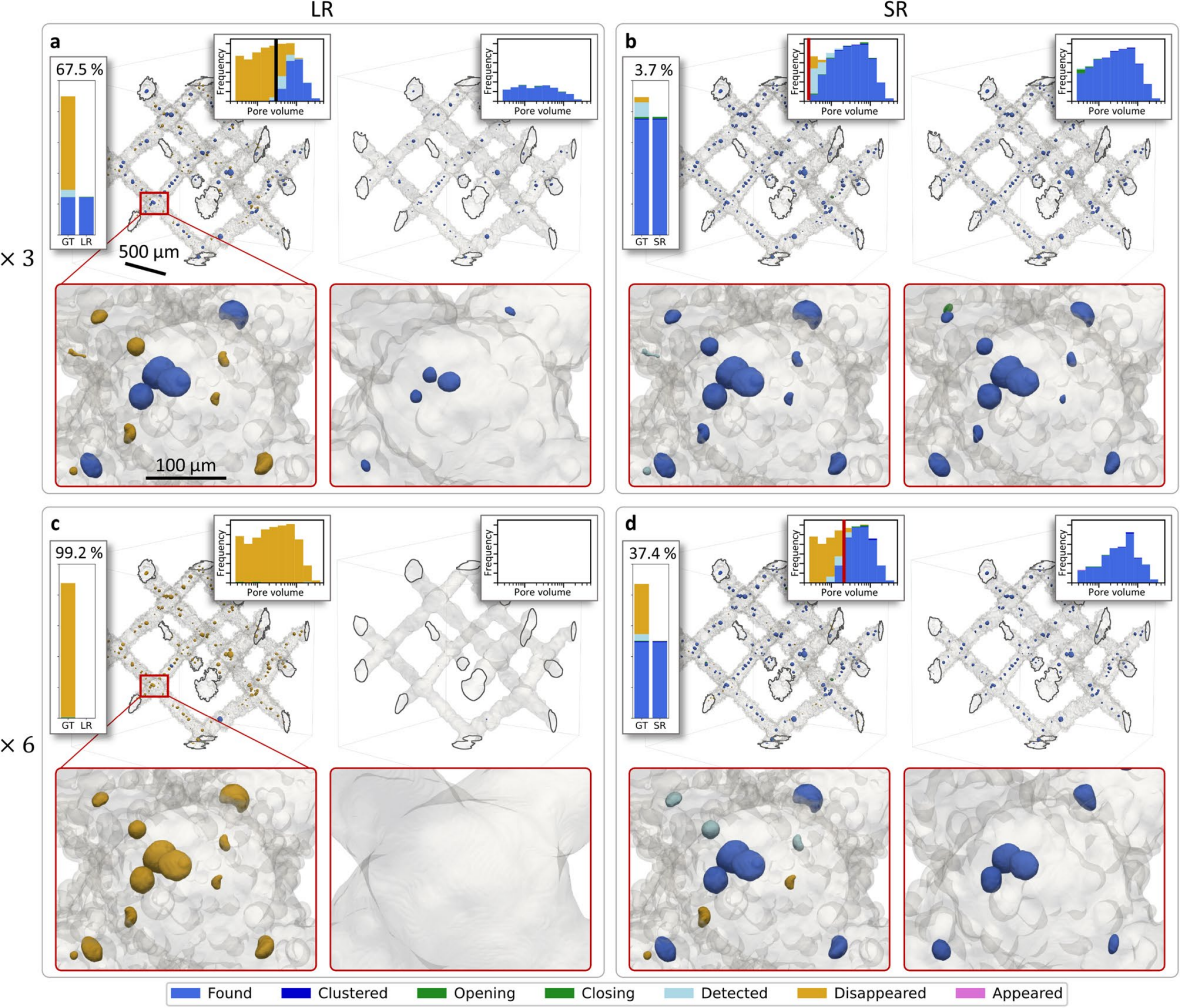
Result of local pairwise pore detection. (**a**) (*top*) 3D renderings of a lattice cell showing the result of the pairwise pore detection applied between GT and LR-3 (*left*). The lattice material is rendered in grey with a transparency to display the pores coloured according to their status. The darker contours represent the ROI boundaries that intersect with the lattice. (*bottom*) Zoomed-in inset for each rendering. (*bar plot*) Pore status distribution, between GT and LR-3. *y*-axis is between 0 and 1,000. Percentage on the top of each bar plot stands for the rate of “*Disappeared*” pores. (*histograms*) Pore volume distribution according to their computed status, between GT and LR-3. The red vertical line denotes the volume of one voxel in the original low-resolution sampling. The black one denotes 300 voxels in volume. 10 logarithmic bins were used to compute each histogram. Pore volume scale is in logarithmic scale and between 20 and 5,000 voxels. Frequency scale is between 0 and 130. (**b**) Same layout but between GT and SR-3. (**c**) Same layout but between GT and LR-6. (**d**) Same layout but between GT and SR-6.

Segmented pores were analysed with a custom pairwise object detection algorithm, with the result presented in Fig. 6. Qualitatively, the majority of the pores (67.5%) were lost in LR-3 (see Fig. 6a), and the morphology of recovered pores was strongly modified. In LR-6, all but two pores were lost, leading to 99.2% of “*Disappeared*” pores, making it impossible to complete further analysis (see Fig. 6c). These observations are completed for each case (LR-3, LR-6, SR-3, SR-6) by the quantification of pore volume distribution according to their computed status. In LR-3, it clearly appeared that the smaller pores mostly disappeared and pores smaller than 300 voxels were systematically lost (see black vertical line in Fig. 6a).

On the contrary, most of the pores in SR-3 were recovered with only 3.7% of “*Disappeared*” pores, and their morphology seemed mostly unaltered (see Fig. 6b). Astonishingly, more than 60% of pores were recovered in SR-6 (see Fig. 6d) and the histogram in Fig. 6c–d denotes that only the larger pores are in fact retrieved. Also, their morphology seemed to have been strongly smoothed out. The threshold volume at which pores are generally recovered in super-resolution was found to match approximately the volume of one voxel in the original low-resolution sampling, *i.e.* $3^3=27$ vx in SR-3 and $6^3=216$ vx in SR-6 (see red line in Fig. 6b,d). Hence, this approach highlighted that the presented super-resolution procedure could be able to retrieved objects as small as one voxel in the original sampling, which were not detected otherwise. The distinction between the status was made to avoid quantification error on local morphology due to splitting or connecting pores (*Clustered*), and to not exaggerate the amount of “*Disappeared*” pores because of the detection threshold (*Detected*) and local surface changes (*Opening* and *Closing*). Furthermore, only very few (two) and small *Appeared* pores were created in SR-6 (see Fig. 6b,d). This finding suggests that the neural network performed well in refining the resolution to uncover small pores and did not learn to reproduce a statistical distribution of pores within the material. It is important to note that despite a significant resolution gap, limited training data and visible streaking artefact, the network generated very few hallucinations.

**Fig. 7:**
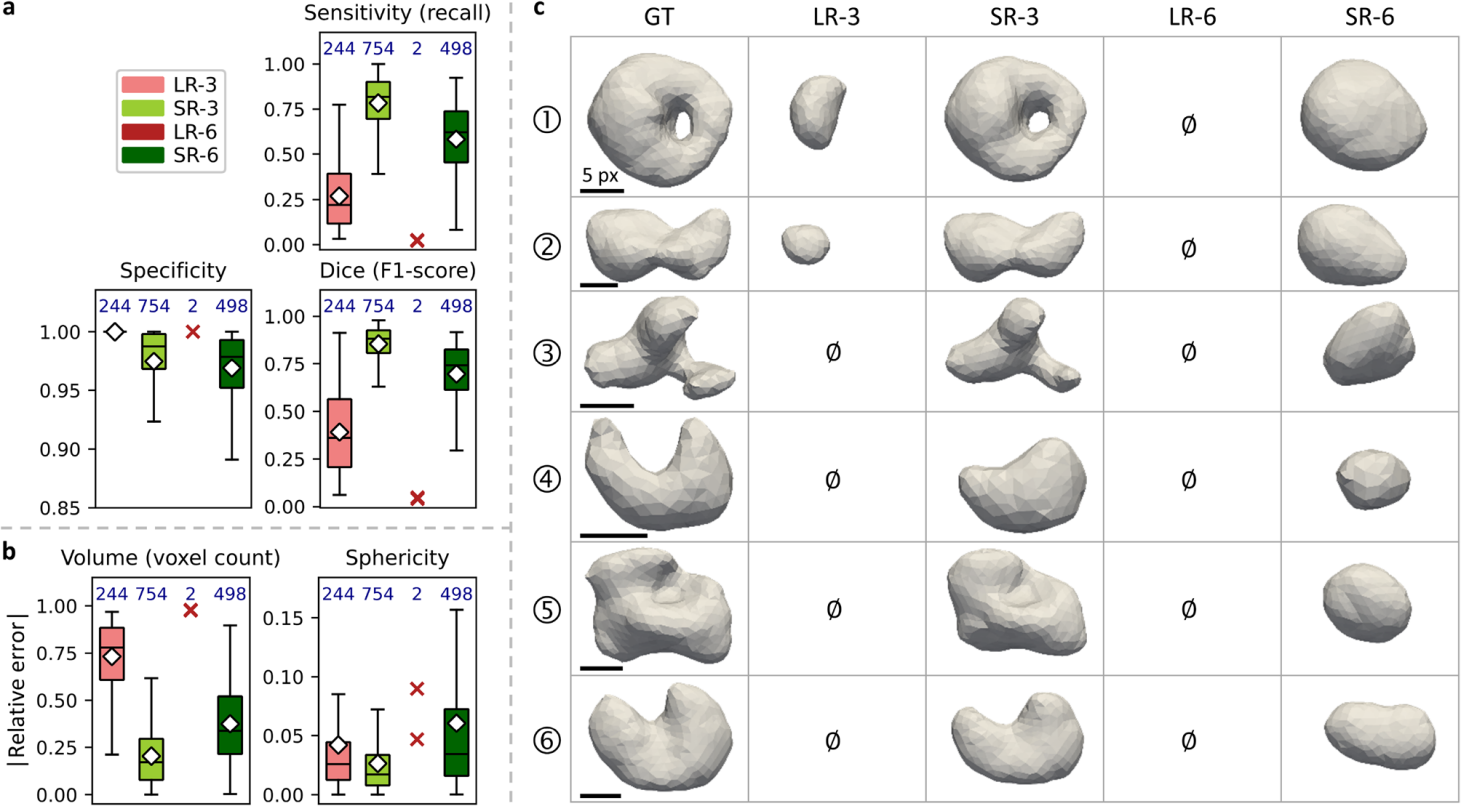
Result of local performance and morphometric measurements. (**a**) Boxplots of performance metrics with GT as the reference. The number at the top of each boxplot corresponds to the number of included pores. Red crosses denote individual points in the case for LR-6 because boxplot depiction was not applicable. White diamonds represent mean values. Outliers are not displayed for visualisation purposes. (**b**) Same layout but displaying the error made on local morphological metrics. (**c**) 3D renderings of six individual pore selected for their relative shape complexity. $\emptyset$ stands for the absence of pore.

Local pairwise pore detection also made possible to compare changes in pore delineation and morphology when processed by the super-resolution procedure. Thus, local performance metrics such as the sensitivity (true positive rate), the specificity (true negative rate) and the Dice score were measured and the result is displayed in Fig. 7a. On the one hand, a sharp increase in sensitivity between LR-3 and SR-3 denotes that fewer voxels were classified as false negative. On the other hand, the slight decrease in specificity is caused by more false positive voxels. It should be noted that the comparison between LR-6 and SR-6 was not possible due to the only two pores found in LR-6. Figure 7b depicts change in the error made on morphometric descriptors such as the pore volume and sphericity.

The super-resolution applied on LR-3 enabled to decrease the error made on pore volume from about 70 % to 20 % in average, and down to around 40 % for SR-6. In most cases, this evolution in fact corresponds to an underestimation of the pore volume. The trend is less obvious for the sphericity, for which the error was already under 10 % in average. This could be explained by the fact that most of the pores were already highly spherical (sphericity $\approx 0.8$, see Fig. 4c) and that the smoothing induced by the super-resolution cancelled out the effective gain in resolution. To go further, six pores displaying a more complex shape than the others were selected and are shown in Fig. 7c. In LR-3, they were generally not found, or with a very altered shape and volume, whereas they were obviously not found at all in LR-6. However, SR-3 allowed to recover successfully the complexity of the shape with a very slight smoothing. Regarding SR-6, all selected pores were found with a reasonable apparent volume, although strongly smoothed out.

**Fig. 8:**
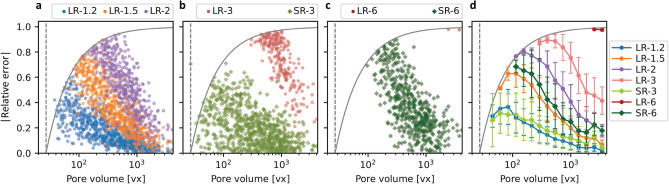
Comparison between low, super and intermediate resolution cases regarding the distribution of the error made on the pore volume according to the pore volume itself in the GT. (**a**) Intermediate case LR-1.2, LR-1.5 and LR-2 with pixel sizes of 3.6 ${\upmu }$m, 4.6 ${\upmu }$m and 6 ${\upmu }$m, respectively. The dotted vertical line corresponds to the detection threshold $\alpha$ defined at 27 voxels, and the solid curve corresponds to this same threshold taking into account the variation of the pore volume (curve equation: $y=-27/x+1$). The x-axis scale starts at 20 voxels. (**b**) Cases LR-3 and SR-3. (**c**) Cases LR-6 and SR-6. (**d**) For all cases, average error made on the pore volume in the GT as a function of the pore volume, calculated within 15 logarithmic bins. Error bars display the standard deviation for each bin.

With the aim to measure the absolute performance of the super-resolution workflow, a comparative study was performed with three CT-scans made at intermediate pixel sizes: 3.6 ${\upmu }$m, 4.6 ${\upmu }$m and 6 ${\upmu }$m named respectively LR-1.2, LR-1.5 and LR-2, in accordance with the established nomenclature. Figure 8a–c represents the distribution of the absolute error made on the pore volume according to the pore volume itself in the GT, for LR-1.2, LR-1.5 and LR-2 in Fig. 8a, for LR-3 and SR-3 in Fig. 8b and for LR-6 and SR-6 in Fig. 8c.

Note that the dotted vertical line corresponds to $\alpha$, the detection threshold defined at 27 voxels, and the solid curve corresponds to this same threshold taking into account the variation of the pore volume (curve equation: $y=-27/x+1$). Thus, any point above this solid line represents necessarily a pore that increased in volume. For all cases, the error made on the volume was found to generally decline with the volume in the GT. In order to compare the different point clouds, Fig. 8d displays the average error made on the pore volume as a function of the pore volume in the GT. This representation confirms the point cloud trends and highlights close matches between SR-3 and LR-1.2, and between SR-6 and LR-1.5. In other words, the error made on pore volume for SR-3, resp. SR-6, is in the same range as for LR-1.2, resp. LR-1.5, for the detected pores, despite a magnification ratio of 2.5, resp. 4, between the initial pixel sizes.

Moreover, it is known that the segmentation procedure has a major influence on the obtained measurements^56^, so the impact of the threshold used for the segmentation of the GT was evaluated. Following the same analysis, the high-resolution scan was segmented using four other automatic thresholding methods commonly available on image processing software such as *Fiji*^57^: Li, Intermodes, Huang and Minimum. These methods were specifically chosen because they enabled convincing delineation between the material and the pores. Then, the segmentations were compared to the Otsu’s one (GT) and SR-3, and the result is displayed in Supplementary Fig. S7. The analysis shows that the error caused by the segmentation itself and the error caused by the super-resolution SR-3 are in the same order of magnitude, which tends to demonstrate the efficiency of the workflow by putting the error generated by the SR method into perspective.

All measurements confirmed that the super-resolution procedure enhanced the image quality and the accuracy of individual pore morphological measurements. Unsurprisingly, it also emphasised the better results obtained with a smaller magnification factor between the GT and the low-resolution image. In this study, smaller pores were mostly recovered with a magnification factor $\times 3$, and their shape (volume, sphericity) was effectively characterised even for the more complex ones. Higher magnification factor ($\times 6$) did not reach the same performance but it enabled lower-level analyses such as the estimation of pore volume distribution with a detection threshold approximately equal to the initial low-resolution pixel size.

### Super-resolution effect on roughness measurements

**Fig. 9:**
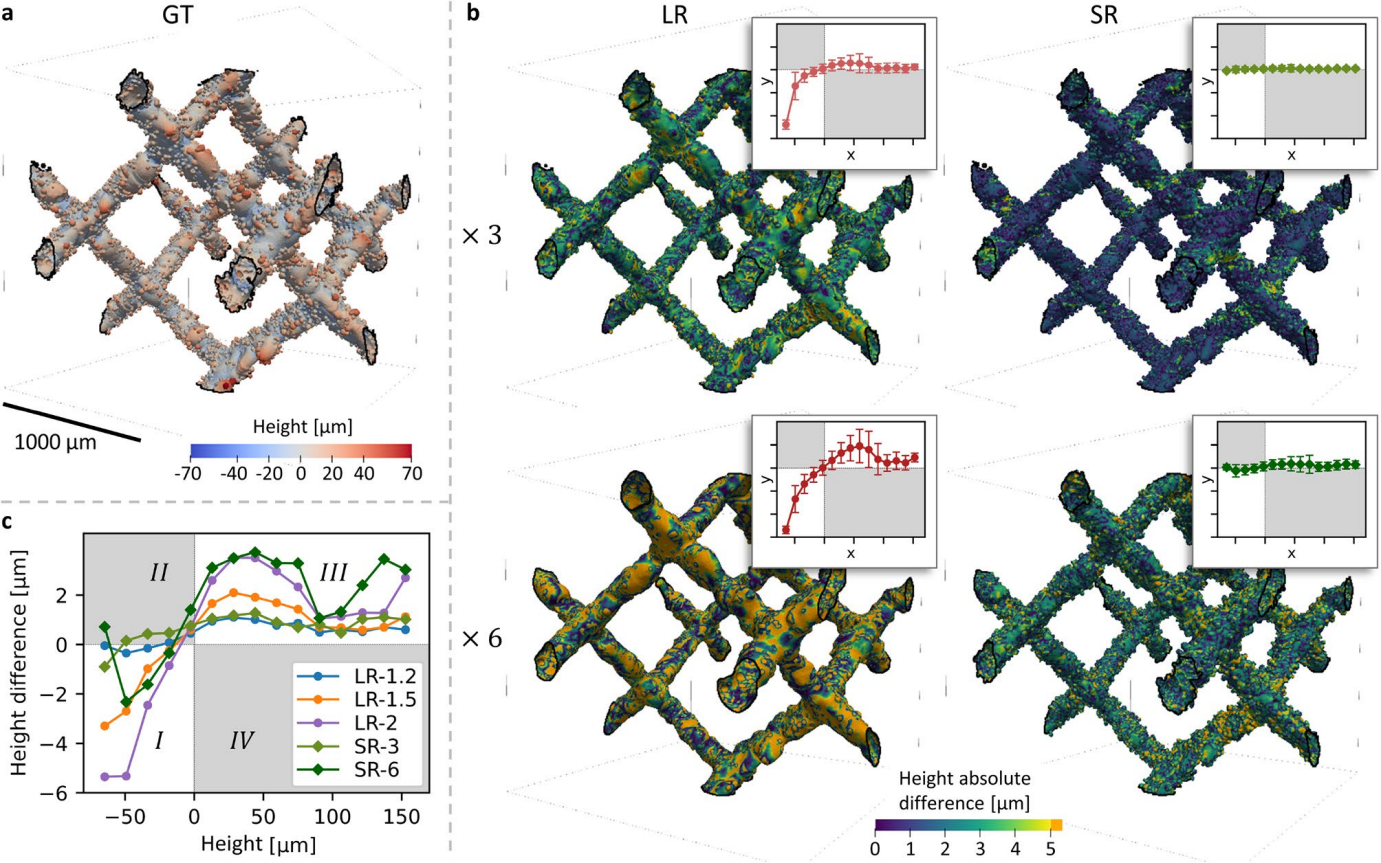
Result of local roughness measurement. (**a**) 3D rendering of a lattice cell displaying the GT surface roughness. (**b**) (*left*) 3D renderings of lattice cells displaying the surface roughness for LR-3 and LR-6, mapped with height absolute difference according to the height measured in the GT. Note that the colour scale saturates in orange above 5 ${\upmu }$m. (*right*) Same layout for SR-3 and SR-6. (*plots*) Mean height difference as a function of the height measured in the GT, calculated within 15 bins. Error bars display the standard deviation for each bin. *x*-axis is height in the GT between -80 and 170 voxels and *y*-axis is mean height difference between -60 and 40 ${\upmu }$m. (**c**) Same layout for LR-1.2, LR-1.5, LR-2, SR-3 and SR-6. Note that the y-axis is zoomed in, and that error bars, LR-3 and LR-6 are not displayed in c for improved visibility.

In addition to roughness parameters measured globally (see Fig. 5e), the surface roughness was analysed locally. Figure 9a shows a 3D height map of the GT surface where height maximums in red are clearly related to partially melted powder. This map was compared with the height maps of LR-3 and LR-6 (see Fig. 9b, left panel), and SR-3 and SR-6 (see Fig. 9b, right panel). The result displays a smoothing of the surface, especially pronounced for LR-6 where surface peaks are planed off and valleys filled (see orange regions). Also, it indicates a decrease of the local height error for super-resolution images, illustrated by the darkening of the maps.

Notches were considered to be critical defects, as they can promote crack initiation and are therefore detrimental for the lattice mechanical properties. Hence, Fig. 9b–c shows height differences according to the height measured in the GT, in order to discriminate between the errors made on peaks and those made on valleys. Four different domains have been identified on such graph: (*I*) valleys are underestimated and filled; (*II*) valleys are overestimated; (*III*) peaks are underestimated and planed; (*IV*) peaks are overestimated. Very few data points were measured in the domains *II* and *IV* (see grey areas in Fig. 9b–c). Moreover, the result highlights that height difference is greatly reduced with super-resolution, especially in the domain *I* and with a lesser extent in domain *III*, compared to LR-3 and LR-6. Comparison of the result with intermediate resolutions emphasises close matches between SR-3 and LR-1.2, and between SR-6 and LR-2 with even a better recovering of the deepest notches for SR-6 (see Fig. 9c). The super-resolution outputs SR-3, resp. SR-6, allow to greatly recover the critical notches, producing height differences similar to LR-1.2, resp. LR-2, despite a magnification ratio of 2.5, resp. 3, between the initial pixel sizes.

As with the pore morphology metrics, local roughness measurements demonstrated the effectiveness of the presented method. Super-resolution images produced high fidelity roughness maps and were especially relevant for critical defects such as deep notches.

### Influence of the multi-resolution registration

**Fig. 10:**
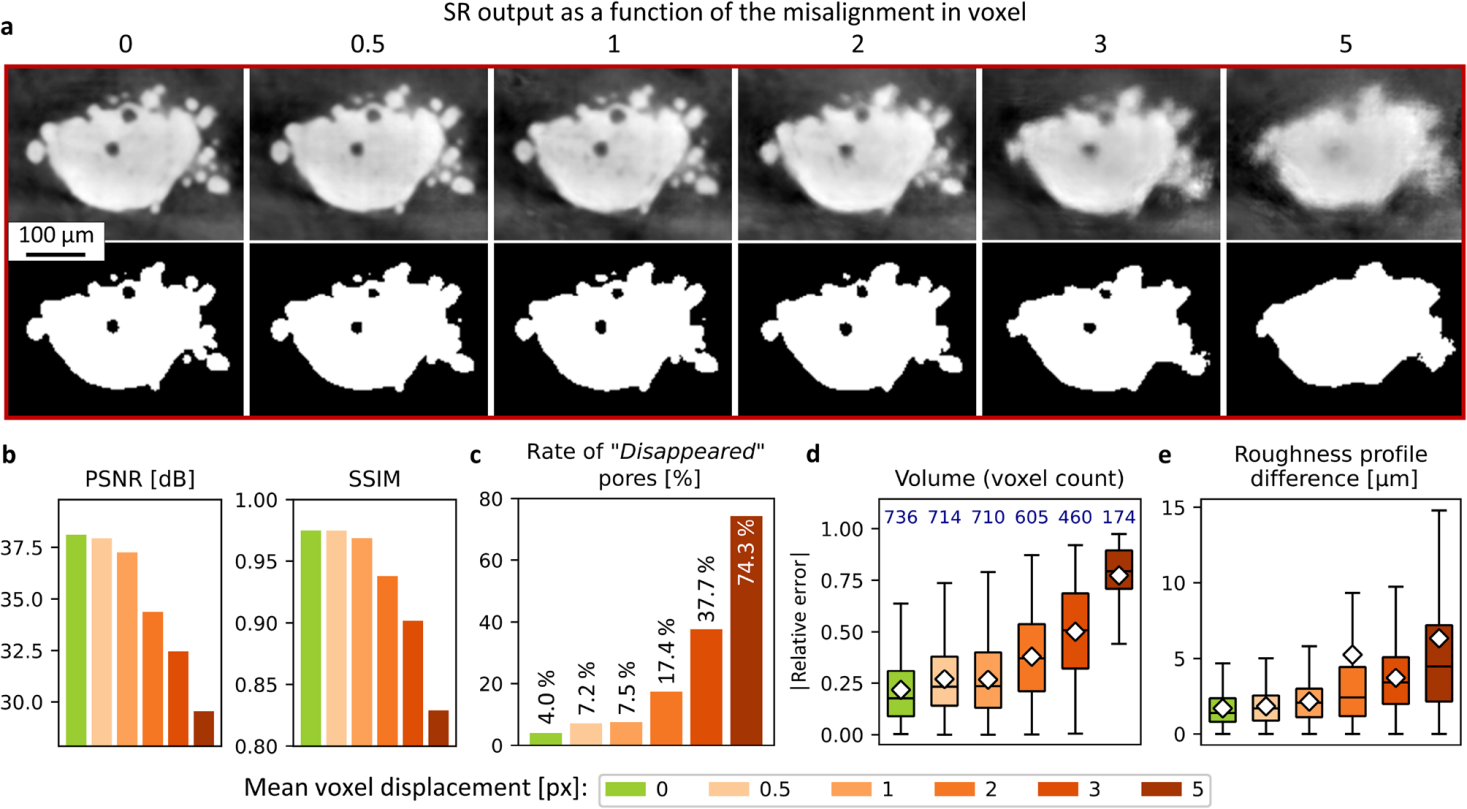
Result of registration sensitivity on SR-3. (**a**) Zoomed-in insets of horizontal slices and segmentations of super-resolution outputs trained with increasingly deformed GT. Image contrast was extended with minimum and maximum greylevels for better visualisation. (**b**) Global greyscale similarity measurements with GT as reference. (**c**) Rate of “*Disappeared*” pores. (**d**) Boxplots of the error made on the pore volume. The number at the top of each boxplot corresponds to the number of included pores. White diamonds represent mean values. Outliers are not displayed for visualisation purposes. (**e**) Same layout but displaying the height absolute difference with the 3D height map of the GT as reference.

The multi-resolution registration is a crucial step of the presented workflow. The sensitivity to registration misalignment was evaluated by applying small transformations to the GT. This misalignment was quantified by calculating the mean voxel displacement, and was set within a range of 0 to 5 px. Then, biased trainings were applied to LR-3 and the result is presented in Fig. 10. For more details about training, evolution of the loss function is given in Supplementary Fig. S2. Zoomed-in slice insets in Fig. 10a exhibit increasing blur with the mean voxel displacement especially visible at material boundary, and most of the details are lost at a displacement of 5 px. However, it is difficult to clearly discriminate qualitatively the best image quality up to a displacement of 1 px. As before, quantitative metrics were calculated between the GT and the biased super-resolution images on greyscale data at global scale (see Fig. 10b), on segmented data at local scale (see Fig. 10c–e). In all cases, the result confirms the observations with a strong decrease of PSNR and SSIM (see Fig. 10b), and an increase of the error made on pore volume and 3D roughness profile (see Fig. 10d–e), for a mean voxel displacement greater than 1 px. Below this value, measurements are quite similar, although global greyscale quality metrics tends to decrease slightly with displacement. The rate of “*Disappeared*” pores to the total number of pores detected is also increasing and about doubling each one pixel of displacement (see Fig. 10c), which highlights a loss of image quality. Thus, this study clearly demonstrates that a high level of registration accuracy – *i.e.* mean voxel displacement below 1 px in the GT sampling – is required for the quality of the super-resolution output.

### Estimation of scan time, storage and energy savings

One of the main objectives of this work was to evaluate the improvements capabilities of the presented super-resolution workflow. Table 2 synthesises savings in scan time, data storage and energy when using the workflow for the presented data with $\times 3$ or $\times 6$ magnification factor. The calculations are detailed in *Methods*. The estimation findings indicate considerable reduction of scan time by a factor of 15, resp. 125, for $\times 3$, resp. $\times 6$ factor. Such difference is due to the cubic evolution of the equivalent tile scan (full FOV) in regards to the magnification factor. Of course, gains must be weighed against the loss of information related to the use of super-resolution and the fact that training time is not taken into account in this case. Indeed, any saving is only effective if the resulting data quality enables the required analysis. For example, as shown in Fig. 6d, counting pores greater than $(6\times 3)^3 = 5832$ ${\upmu }\hbox {m}^{3}$ could be done with SR-6 with little error, while it is not possible to measure their volume with an error of less than 40 % (see Fig. 7b). With this in mind, the potential reduction in scan time is still tremendous and can be a real game-changer for such multiscale structure. This is especially true for synchrotron imaging where beam time is valuable and long scans must be avoided, as well as for achieving high-throughput characterisation of materials.

Besides, data storage savings about 50 % are lower because they are only due to the absence of projection data for super-resolution outputs. Energy savings result from scan time savings, and reach reduction of consumption by a factor of 2, resp. 11, for $\times 3$, resp. $\times 6$ factor. They are mitigated by the consumption of deep learning computations, which are not negligible. In the future, direct measurement of energy consumption of graphic units will be available on the cluster used in this study, enabling more accurate estimates of energy savings. To sum up, the proposed methodology could reduce scan time, and with a lesser extent data storage and energy consumption, which is of great interest for both research and industrial applications.

**Table 2:** Estimation of scan time, storage and energy savings with SR-3 and SR-6.

	Scan time [s]			Data storage [GB]			Energy [kWh]		
	Original	Super-resolution	Evolution	Original	Super-resolution	Evolution	Original	Super-resolution	Evolution
Factor $\times 3$	58, 042	3, 703	$\div 15$	324	181	$\div 2$	81	32	$\div 2$
Factor $\times 6$	464, 335	3, 703	$\div 125$	2, 589	1, 402	$\div 2$	645	61	$\div 11$

Displayed values have been rounded.

## Conclusion

The presented super-resolution methodology based on an MSD neural network^47^ has proved its effectiveness for improving defects detection and measurement by enhancing the quality of low-resolution images of a steel lattice. This enhancement was not only demonstrated using global visual similarity metrics, but above all directly on local metrics on features of interest: pores and surface roughness. It allowed to recover most of the metrics of interest with little change on their values, which was not at all possible from the low-resolution images. Even complex pore shapes and deep surface notches were accurately retrieved.

Also, two magnification factors between low and high-resolution images were investigated in this study. It was deduced that the ratio between the magnification of ground truth and low-resolution should not be considered as the factor limiting the improvement in image quality. Instead, the size of the smallest feature of interest (*i.e.* 27 voxels at high-resolution or 729 ${\upmu }\hbox {m}^{3}$) could be a good indicator for the choice of the low-resolution pixel size, as it has been shown that most defects smaller than one voxel in the low-resolution image are not found in the super-resolution image. An emphasis was also placed on the impact of the accuracy of the prior multi-resolution registration, showing the importance of this step.

Compared to other strategies for reducing scan time such as sparse-view reconstruction algorithm or denoising, this super-resolution method also takes advantage of the need to acquire an initial high-resolution scan. Indeed, as shown in this study, this “ground truth” scan can be used both for feeding the neural network and to estimate the measurement uncertainties. Hence, the reduction in scan time enabled by super-resolution can reach several order of magnitude while assessing the impact on defect quantification.

This work opens the way to a number of methodological perspectives and research applications. Further studies should provide a better understanding of the impact of the neural network architecture and the limits concerning the generalisation of the model. The proposed methodology could also be applied for the study of other types of defects such as cracks, which can be very challenging to detect at limited spatial resolution^39^, particularly because of their anisotropy. Furthermore, it could be extended to other materials and multi-resolution imaging techniques, for example to enhance image quality of hierarchical phase-contrast tomography of human organs^58^.

## Supplementary Information

Supplementary Information.

## Data Availability

The dataset supporting the main findings of this study is publicly available on the recherche.data.gouv.fr repository under the open license Etalab 2.0, which permits use, sharing, and adaptation with appropriate attribution. It can be accessed directly via the following 10.57745/VDKKAH. The dataset includes the training, validation and test datasets used for deep learning as well as the masks used both for the training and for the global quantification of the image quality. Detailed metadata and documentation accompanying the dataset are also available on the repository. Other datasets generated and/or analysed during the current study as well as the Python code for multi-resolution registration and pairwise object detection are available from the corresponding author upon request.
