# Supplementary material for: Super-resolution X-ray tomography using deep learning applied to the 3D quantification of defects in lattice structures
Source: Sci Rep. 2025 Oct 21;15:36664. doi: 10.1038/s41598-025-20372-4 (PMC12540691; doi:10.1038/s41598-025-20372-4)
Supplement: Supplementary file 1 — Supplementary Information. [file 41598_2025_20372_MOESM1_ESM.pdf]

Supplementary Information

Super-resolution X-ray tomography using deep learning applied to the 3D quantification of defects in lattice structures

Antoine Klos[1,*], Luc Salvo[1], and Pierre Lhuissier[1]

[1]Univ. Grenoble Alpes, CNRS, Grenoble INP, SIMAP, 38000 Grenoble, France
[*]antoine.klos@simap.grenoble-inp.fr

Contents

Mask **1**
Supplementary Figure S1 1
Loss function **2**
Supplementary Figure S2 2
Pairwise object detection algorithm **2**
Supplementary Note S1 2
Supplementary Figure S3 3
Supplementary Figure S4 4
Supplementary Figure S5 4
Supplementary Figure S6 5
Impact of the pore segmentation **6**
Supplementary Figure S7 6
3D roughness measurements **6**
Supplementary Note S2 6

Mask

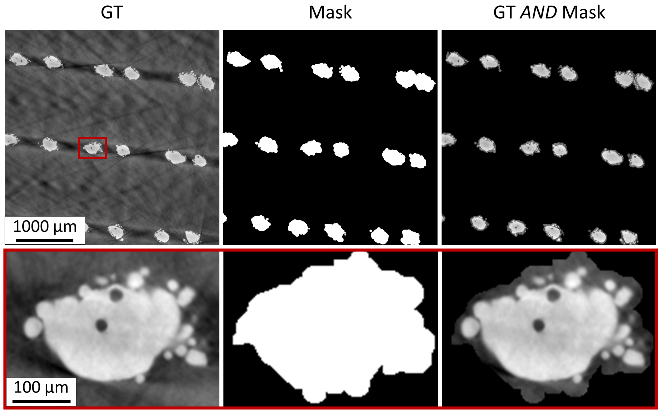

Figure S1. Horizontal slices of high-resolution CT-scan (*left*), of the mask used for greyscale similarity metrics (*centre*), and of the overlay (*right*). An equivalent mask was used for networks training.

Loss function

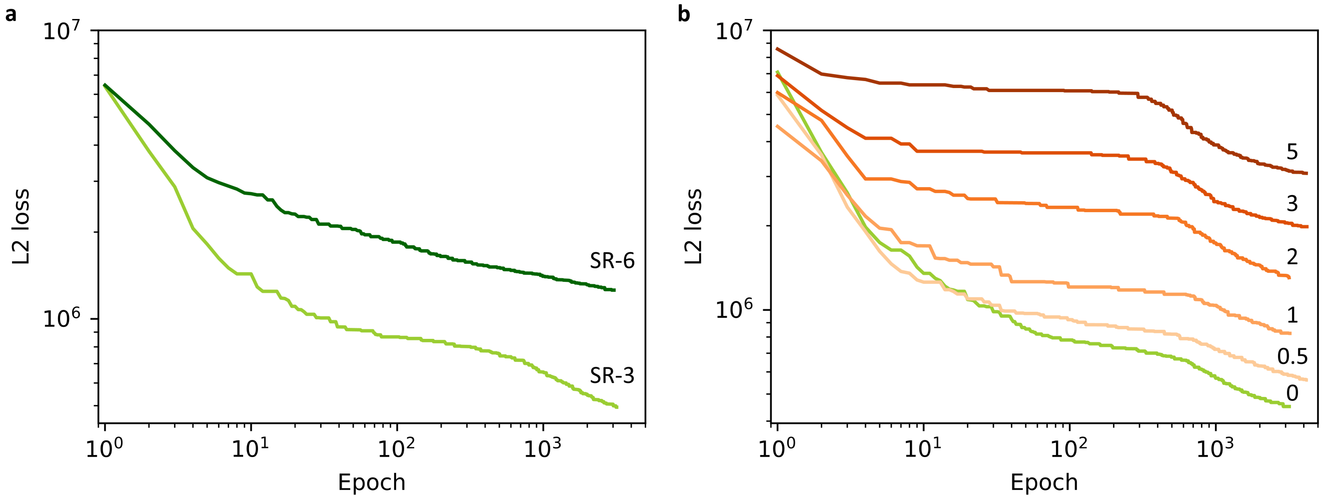

Figure S2. Evolution of the best validation loss during training. **a.** Comparison of SR-3 and SR-6, respectively the super-resolution trainings between the ground truth and the low-resolution scans with pixel sizes of 9 µm and 18 µm. **b.** Comparison of the loss function evolution for registration accuracy evaluation. The mean voxel displacement is expressed in pixel.

Pairwise object detection algorithm

Supplementary Note S1

This note details the definition of the pore/object status and introduces the following figures.

- *Found*: the same object was found in both $V0$ and $V1$;
- *Detected*: the object was not "*Found*" but an overlap large enough existed with an object smaller than the detection threshold;
- *Clustered*: the object was found but it was merged with another object and/or divided into several objects;
- *Appeared*: the object was only detected in $V1$;
- *Disappeared*: the object was only detected in $V0$;
- *Opening*: the object was not "*Found*" but an overlap large enough existed between the object in $V0$ and the outside region of the lattice in $V1$, which could be interpreted as a pore opening;
- *Closing*: the object was not "*Found*" but an overlap large enough existed between the object in $V1$ and the outside region of the lattice in $V0$, which could be interpreted as a pore closing.

A "large enough" overlap was defined as an overlap at least half the size of the object of interest, *i.e.* $t = 0.5$ in Figure S3b. Details about the pairwise object detection algorithm logic can be found in Figure S3. In-depth examples of the algorithm behaviour are shown in Figure S4 and Figure S5 for specific cases. Finally, the validation of the algorithm is presented in Figure S6. A phantom that included 25 typical cases encountered within the lattice was tested, and results met expectations.

a

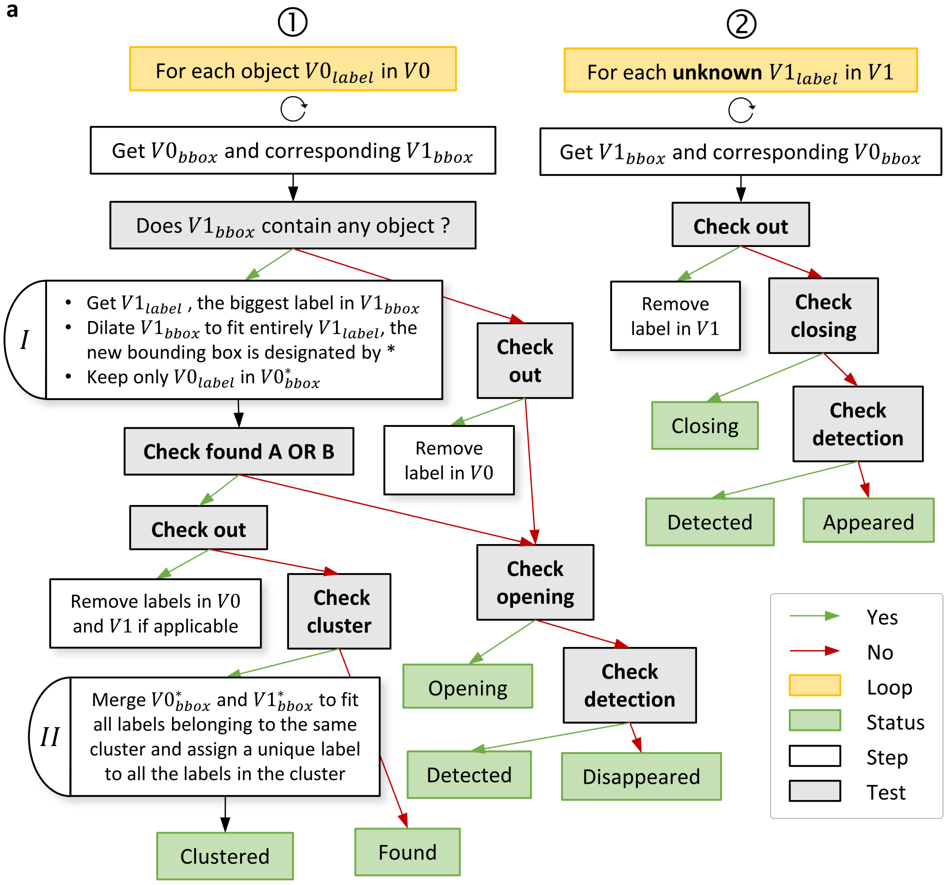

b

**Check out**	Is the bounding box located at a boundary of the volume ?
**Check found A**	For each object in $V1^{*}_{bbox}$: is its centroid within the convex hull of $V0_{label}$ in $V0^{*}_{bbox}$ **AND** is the object overlap with $V0_{label}$?
**Check found B**	Is the centroid of $V0_{label}$ in $V0^{*}_{bbox}$ within the convex hull of the biggest object's in $V1^{*}_{bbox}$ **AND** is the object overlap with $V0_{label}$?
**Check opening or closing**	Is there an overlap between the label and the outside region of the lattice which is large enough relative to the label volume ?<br>• opening: $V0_{label} \cap NOT(V1_{lattice}) > t \times V0_{label}$<br>• closing: $V1_{label} \cap NOT(V0_{lattice}) > t \times V1_{label}$
**Check detection**	Is there any object smaller than the volume threshold which overlaps with the label **AND** whose overlap is large enough relative to the object volume ?<br>a) $V0_{label} \cap V0_{object} > t \times V0_{object}$<br>b) $V1_{label} \cap V1_{object} > t \times V1_{object}$
**Check cluster**	Do several object within the bounding box pass the previous tests **OR** any of these objects' labels is already known ?

Figure S3. **a.** Block diagram of the algorithm for pairwise object detection. Steps I and II are detailed in Figure S5 with specific examples. Algorithms ① and ② are executed sequentially. **b.** Detailed description of the tests performed within the algorithm. Some tests are illustrated in Figure S4. In the study, t was set to 0.5.

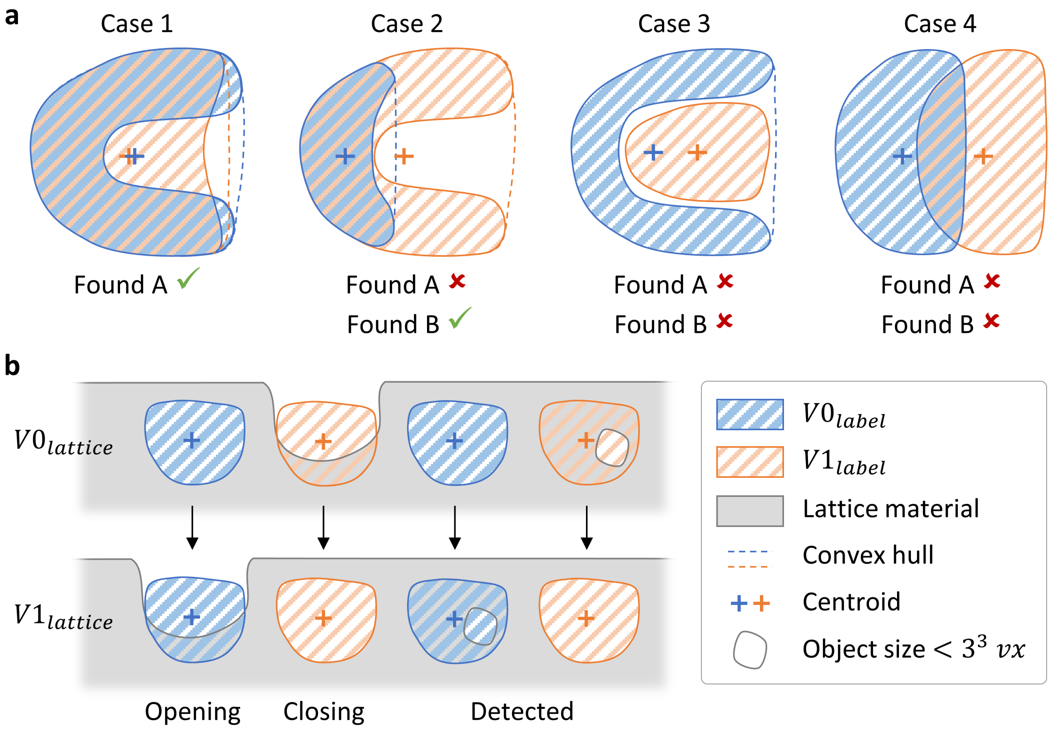

Figure S4. 2D example of the algorithm behaviour in the case of found, opening, closing, and detected objects. **a.** Scheme of different cases were an object status is considered to be found or not by the pairwise object detection algorithm. In case 1 and 2 the objects are matched (status *Found*) whereas in case 3 and 4, the objects are considered not close enough to be matched, according to the tests defined in Figure S3b. **b.** Scheme of cases for which the object status could be set to *Opening*, *Closing* or *Detected* according to the tests defined in Figure S3b.

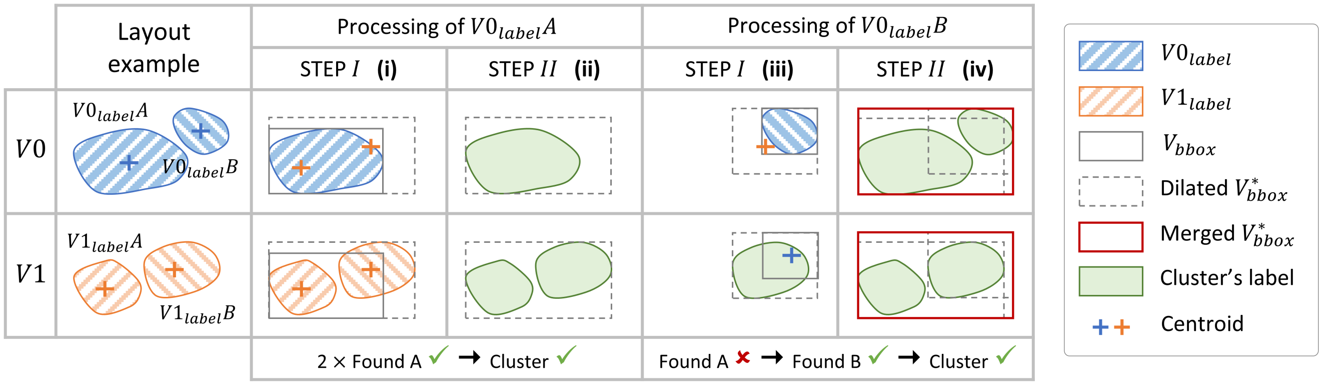

Figure S5. 2D example of the algorithm behaviour in the case of a cluster of objects. (i) Dilate $V0_{bbox}A$, *Check cluster* raise that several objects within $V1^*_{bbox}A$ are found ($V1_{label}A$ and $V1_{label}B$); (ii) Assign a unique label to all objects in the cluster; (iii) Dilate $V0_{bbox}B$, an object ($V1_{label}B$) is found within $V1^*_{bbox}B$ and *Check cluster* raise that this object is already known; (iv) Merge dilated bounding boxes and assign a unique label to all objects in the cluster. Steps I and II correspond to the steps described in Figure S3a

a

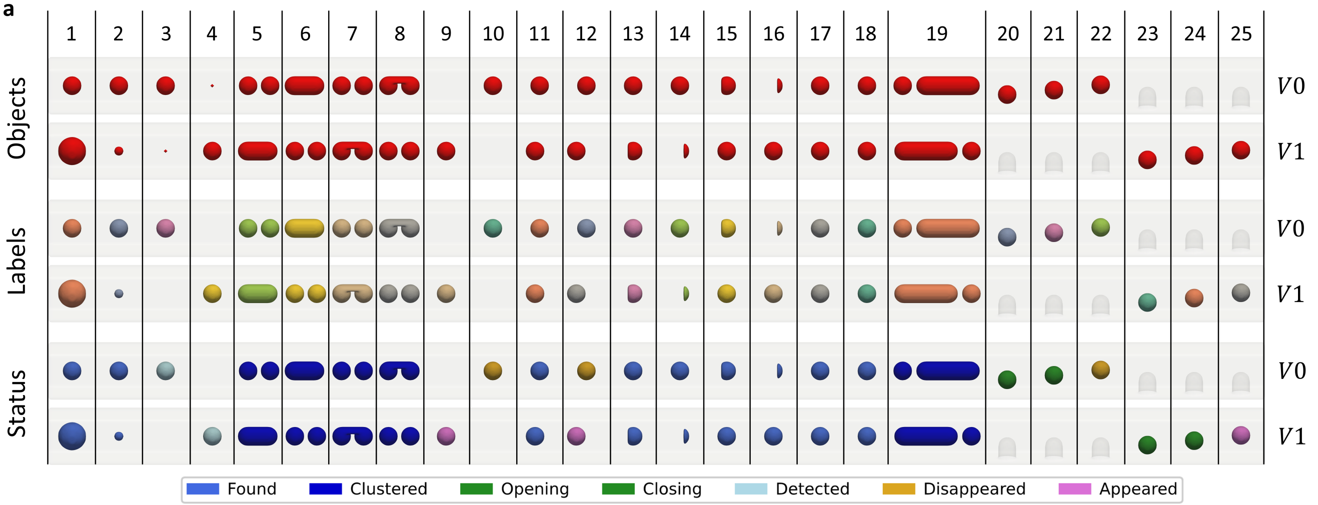

b

1. Pore volume increases between $V0$ and $V1$. → *Found*
2. Pore volume decreases between $V0$ and $V1$. → *Found*
3. Pore volume decreases between $V0$ and $V1$ to below the detection threshold. → *Detected*
4. Object volume increases between $V0$ and $V1$ until it exceeds the detection threshold. → *Detected*
5. (A) Formation of a cluster in $V1$ from separate pores in $V0$. → *Clustered*
6. (A) Splitting of a cluster in $V0$ into separate pores in $V1$. → *Clustered*
7. (B) Formation of a cluster in $V1$ from separate pores in $V0$. → *Clustered*
8. (B) Splitting of a cluster in $V0$ into separate pores in $V1$. → *Clustered*
9. Appearance of a pore in $V1$. → *Appeared*
10. Disappearance of a pore in $V1$. → *Disappeared*
11. Shifting of a pore in $V1$, the center of mass of the $V1$ pore intersects the $V0$ pore's convex hull and *vice versa.* → *Found*
12. Shifting of a pore in $V1$, the center of mass of the $V1$ pore does not intersect the $V0$ pore's convex hull and *vice versa.* → *Dis/appeared*
13. (A) Change of pore shape between $V0$ and $V1$. → *Found*
14. (B) Change of pore shape between $V0$ and $V1$. → *Found*
15. (C) Change of pore shape between $V0$ and $V1$. → *Found*
16. (D) Change of pore shape between $V0$ and $V1$. → *Found*
17. Solid pore in $V0$ becomes hollow in $V1$. → *Found*
18. Hollow pore in $V0$ becomes solid in $V1$. → *Found*
19. Formation of a complex cluster in $V1$ from and into separate pores in $V0$. → *Clustered*
20. (A) Pore opening with a large enough overlap between the pore in $V0$ and the outside region of the lattice in $V1$. → *Opening*
21. (B) Pore opening with a large enough overlap between the pore in $V0$ and the outside region of the lattice in $V1$. → *Opening*
22. Pore opening with a too small overlap between the pore in $V0$ and the outside region of the lattice in $V1$. → *Disappeared*
23. (A) Pore closing with a large enough overlap between the pore in $V1$ and the outside region of the lattice in $V0$. → *Closing*
24. (B) Pore closing with a large enough overlap between the pore in $V1$ and the outside region of the lattice in $V0$. → *Closing*
25. Pore closing with a too small overlap between the pore in $V1$ and the outside region of the lattice in $V0$. → *Appeared*

Figure S6. Validation of pairwise object detection algorithm on a phantom representing two volumes to compared which includes 25 typical cases. **a.** 3D renderings of both phantom volumes. The lattice material is rendered in grey with a transparency to show the pores. (*top*) All objects, including object smaller than the detection threshold, (*center*) all detected pores, labelled using the algorithm, (*bottom*) all detected pores, coloured by the status using the algorithm. **b.** Description of the 25 typical cases and their associated status given by the pairwise object detection algorithm.

Impact of the pore segmentation

It is known that the segmentation procedure has a large impact on the obtained measurements. Here, the impact of the threshold used for the segmentation of the GT was evaluated. Following the same analysis, the high-resolution scan was segmented using four other automatic thresholding methods: Li, Intermodes, Huang and Minimum. These methods were specifically chosen because they enabled convincing delineation between the material and the pores (see Figure S7a). Then, the segmentations were compared to the Otsu's one (GT) and SR-3, and the result is displayed in Figure S7b. The analysis shows that the error caused by the segmentation itself and the error caused by the super-resolution SR-3 are in the same order of magnitude, which tends to demonstrate the super-resolution efficiency.

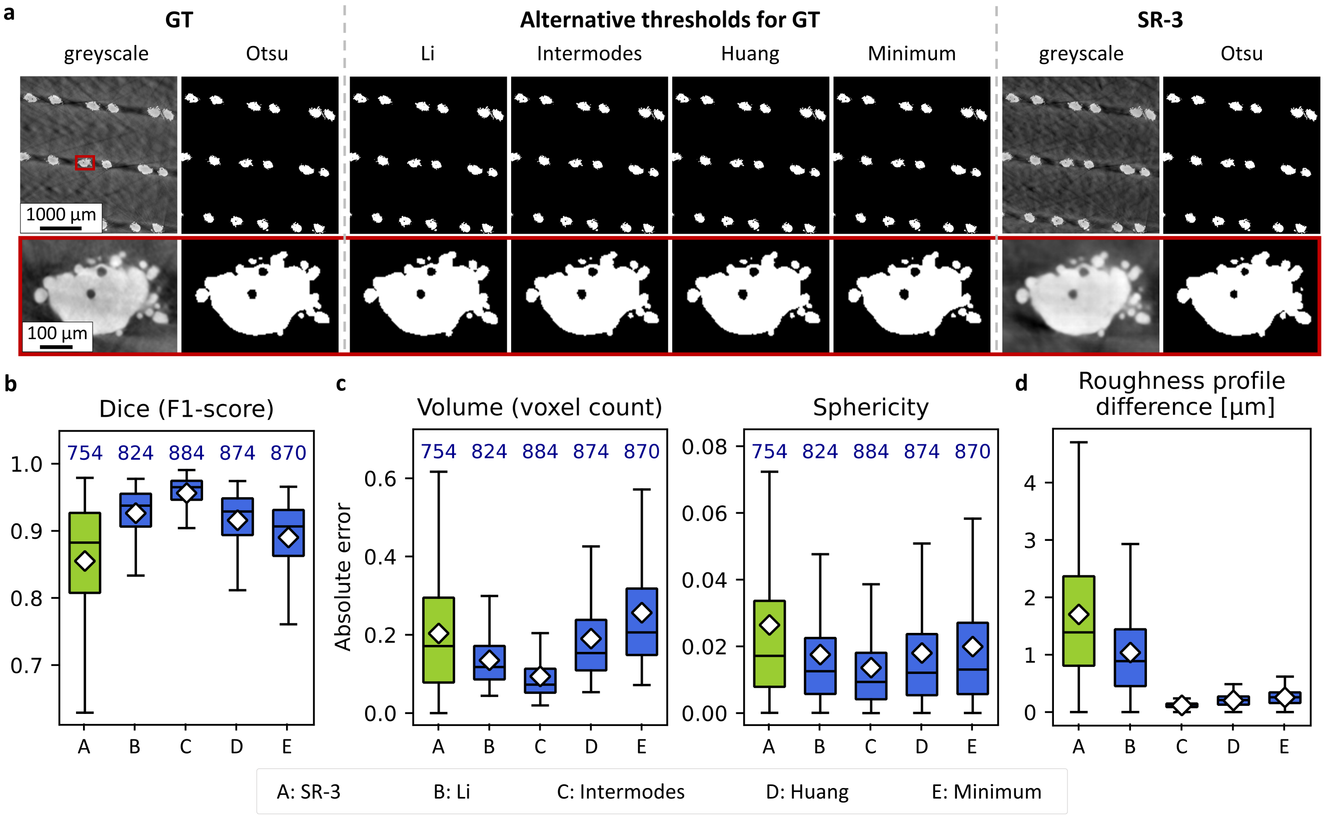

Figure S7. Qualitative and local quantitative assessment of the impact of alternative thresholds on the GT. **a.** (*left*) Horizontal slices of the GT CT-scan and its segmentation with Otsu threshold. (*center*) Horizontal slices of the GT segmentation with alternative thresholds. (*right*) Horizontal slices of the SR-3 super-resolution output and its segmentation with Otsu threshold. when applicable, image contrast was extended with minimum and maximum greylevels for better visualisation. **b.** Boxplots of Dice score performance metric for SR-3 (as reference) and for each alternative threshold. The number at the top of each boxplot corresponds to the number of included pores. White diamonds represent mean values. Outliers are not displayed for visualisation purposes. **c.** Same layout but displaying the error made on local morphological metrics. **d.** Same layout but displaying the height absolute difference between the 3D height map of the GT and each other cases.

3D roughness measurements

Supplementary Note S2

This note details the choice of λ_c, the cut-off wavelength discriminating form and waviness from roughness.
It is advise by the ISO standards (ISO 25178-3:2012) to set λ_c to be five times the size of the largest feature of interest, choosing among a list of predetermined values. In our case, the size of the largest feature of interest is about the size of the steel powder used in the manufacturing process, *i.e.* about 45 µm, which is in line with the selected values of $\lambda_c = 250$ µm.
